# Mothers’ Perspectives: Daily Life When Your Child Has Sensory Differences

**DOI:** 10.1177/15394492241238357

**Published:** 2024-03-19

**Authors:** Susan Allen, Amanda Branson, Shelly J. Lane, Fiona J. Knott

**Affiliations:** 1 University of Reading, UK; 2 Oxford Brookes University, UK; 3 Colorado State University, Fort Collins, USA

**Keywords:** parenting, sensory integration, participation, occupation

## Abstract

A child’s sensory processing and sensory integration (SP-SI) differences can be a barrier to participation in daily life for both child and mother. Supporting mothers is advocated for, but little is known about the everyday experiences of these mothers. To gain an understanding of daily life for mothers and their children with SP-SI differences. Qualitative semi-structured interviews with six mothers were analyzed through thematic analysis. Theme 1 described the impact of child SP-SI on daily life, including challenges in occupations across environments, adaptations required, and the lack of knowledge and understanding from social and professional networks. Theme 2 identified what helps: empowering mothers through relationships based on listening, gaining knowledge, and understanding, and adapting the activity and the environment. Mothers report that their child’s SP-SI differences impact daily occupations and social relationships. In addition, supportive relationships, adapting activities, and adapting the environment, support participation.

## Plain Language Summary

What Is Daily Life Like When You Have a Child With Sensory Processing and Sensory Integration Differences?

To develop a better understanding of everyday life with a child with sensory processing and sensory integration (SP-SI) differences, mothers were asked about their experiences. Six mothers were individually interviewed. They reported challenges to everyday life at home, at school, and in their local communities, for example, sleep, mealtimes, and leisure choices were impacted for both themselves and their child. They faced a lack of understanding from family, friends, and professionals, and had to adjust their activities and routines to accommodate for their child’s needs. It helped mothers to be listened to and to interact with other people who had knowledge and understanding of SP-SI differences. It also helped to be able to adjust activities, routines, and their environment to better suit their child’s sensory needs.

## Introduction

Sensory processing and sensory integration (SP-SI) differences (Watling et al., 2018), also referred to as sensory differences (Royal College of Occupational Therapists, 2021) impact many aspects of children’s participation in everyday activities. Areas affected include play, social participation, activities of daily living, sleep, engagement in learning (Parham & Cosbey, 2019), and shared family occupations (Schaaf et al., 2011). Much of the existing literature focuses on addressing the specific needs of the child, with limited attention paid to the perspective of parents. The focus of this study is the daily life experiences of mothers of children with reported SP-SI differences. Understanding mothers’ experiences will help to inform future strategies for supporting mothers in their daily occupation of parenting.

As occupational therapists, our goal is to enable participation in daily occupations in a way that supports the health and well-being of children and their families (Law, 2002). SP-SI differences are one factor impacting participation in daily occupations, such as mealtimes and homework (Ben-Sasson et al., 2013). The complex interaction between the child and family, and their social and cultural context has not been unpacked in the literature. What we do know is that where child SP-SI differences are reported, investigators often view findings through a deficit lens, identifying elevated levels of parent stress and caregiver strain (Kirby et al., 2019), along with altered family and social relationships, for example, juggling the needs of siblings (Schaaf et al., 2011). Mothers are frequently blamed for their child’s behavior within family and school settings (Chiu, 2013). To shift the narrative and promote participation in daily occupation for children and families following a socioecological perspective (Curtin et al., 2017), a broader understanding of both daily life and the factors that might support mother and child participation is needed.

A number of approaches to working with children with SP-SI differences and their families are available, including environmental adaptation, working directly with the child or working with parents (Reynolds et al., 2017). Individual parent coaching interventions can improve child participation in daily occupations, reduce parental stress, and reinforce a parent’s sense of competence (Miller-Kuhaneck & Watling, 2018). Parents report strategies they have developed themselves to help with everyday life, such as controlling their child’s sensory environment or maintaining a proactive attitude (Schaaf et al., 2011). Mothers also report the importance of continuing an employment role outside the family home (Dunstan & Griffiths, 2008). However, while we know that mothers of children with SP-SI differences face additional demands to those experienced by other mothers, there are few in-depth explorations of their experiences and the strategies they employ.

Thus, the purpose of this study was to examine the experience of daily life for mothers of children with SP-SI differences. Secondarily, we explored what mothers identify as supportive with regard to the impact of their child’s SP-SI differences.

## Method

### Design

It is acknowledged that a wide range of terminology is used in this field. In light of this, and in keeping with person-centered practice, we have chosen to use the phrase “SP-SI differences” in this article. Mothers are experts in their perception of the impact of their child’s SP-SI differences to daily family life. This study uses an inductive approach and qualitative design to understand mothers’ perspectives through one-to-one interviews. Participant involvement was guided in line with CONSORT, the Consolidated Criteria for Reporting Qualitative Research (COREQ) checklist (Booth et al., 2014) and the National Institute for Health briefing (INVOLVE, 2012). Ethical approval was granted through the University of Reading Research Ethics Committee.

### Participants

A purposive community sample (Bowling, 2014) was recruited by convenience through specialist online parent forums in the United Kingdom. No prior contact between researchers and mothers occurred prior to invitation to participate. Inclusion criteria were: mothers who reported SP-SI differences in their children (aged under 19) and resident in the United Kingdom. Exclusion criteria: non-English speakers and, in this exploratory phase, primary carers other than mothers. As an initial exploratory study, a sample size of six or more was identified as appropriate (Braun & Clarke, 2022).

The research team brought a range of backgrounds to this study and all contributed to the study’s research design and data collection. The first author is a female occupational therapist with over 30 years of clinical experience and a postgraduate research student with an interest in parent stress, family support and sensory differences. The second author is an occupational psychology academic and researcher with an interest in learning, motivation and performance. The third author is an occupational therapy academic and researcher with over 40 years of experience and a special interest in sensory integration and neuroscience. The fourth author has over 30 years of experience as a clinical psychologist. She is also an academic with a special interest in autism, anxiety and families.

Fifteen mothers expressed initial interest. Seven completed consent forms but one withdrew from interview due to child illness. Table 1 describes demographics. Pseudonyms are used for confidentiality. All mothers described themselves as white with graduate or postgraduate levels of education. Mothers (*N* = 6) were all in their own homes at the time of interview and either alone or with their direct family. Children (*N* = 10) had been identified as having SP-SI differences by a parent, nurse, or occupational therapist. Mean lifetime access in the United Kingdom (Royal College of Occupational Therapists, 2022) to occupational therapy was 8 hours.

**Table 1.** Participant and Child Demographic Characteristics.

Characteristics	*N* or *M* (*SD*)
Mother	6
Mothers age	45.5 (7.4) years
Highest educational level	
High school or college	—
Bachelor’s degree	1
Master’s degree or doctorate	5
Ethnicity—White	6
Employment	
Employed	4
Self employed	1
Unemployed	1
Child with reported SP-SI differences	10
Age	12.2 (3.6) years
Child gender	
Female	6
Male	4
Child diagnoses	
Autistic spectrum condition or awaiting assessment	6
Developmental coordination disorder or dyspraxia	3
Dyslexia	2
Irlen syndrome	2
Specific language Impairment	2
Selective mutism	1
Slow processing speed	1
Anxiety	1
Learning disability	1
School setting	
Specialist	3
Mainstream	7

*Note*. SP-SI = sensory processing and sensory integration.

### Procedure

Following written informed consent, online or telephone interviews were arranged according to each mother’s preference. Interviews were conducted prior to the COVID-19 pandemic and guided by the evidence base on qualitative interviewing (Howitt, 2010).

The interview guide of semi-structured questions was developed by the authorship team. The guide was reviewed by two community partner mothers: no changes were suggested. Primary guide questions are given in Table 2. It is acknowledged that it can be difficult to identify whether sensory or primary diagnosis factors drive functional difficulties. Mothers were asked to focus on their child’s sensory processing difficulties, rather than difficulties primarily associated with other conditions, such as autism.

**Table 2.** Primary Guide Questions.

Primary guide questions
a. What is life like for you for you with a child with sensory integration difficulties?
b. How does this impact daily life? That is to say, are there things that you or your children are not able to do in everyday life because of their sensory processing and integration difficulties?
c. What has been useful to you? What has not been useful? What support would you like to have had?

Interviews were completed in a single session and their duration varied from 30 to 75 minutes. The first author completed audio recorded interviews. Field notes were taken to support understanding. Probe questions were used as needed to allow mothers to develop their responses more fully, and interviewees were encouraged to add areas of importance to them if these were not already covered in the interview. Recordings were transcribed blindly by a medical secretary and then checked for accuracy by the first author. One participant requested a copy or her transcript. All documentation was anonymized for confidentiality. The first author carried out all interviews. Participants were aware of the interviewer’s occupation and research background, in particular, her interest in mothers’ experience of family life when a child has SP-SI differences driven by the goal of service improvement.

### Data Analysis

Transcriptions were uploaded by the first author into NVivo12 and analyzed using the six-phase framework of thematic analysis (Braun & Clarke, 2022) process. These phases involve familiarizing yourself with the data set, data coding, initial theme generation, theme defining refining, theme naming, and write up. To support reflexivity (acknowledging the researcher’s role), all four researchers considered codes and themes. Coding was latent, with meaning being sought within content. Themes and subthemes were reviewed and refined by all four authors to ensure the thematic structure provided a systematic interpretation of the data (data available on request from first author). Theoretical sampling requires sampling and analysis of data until no new codes are identified and conceptualization is well-developed. The content of the data related closely to the researchers’ questions and supported the sample size. Themes were consistent across interviews, although it is acknowledged that in interpreting the meaning of data, it is never possible to reach an absolute endpoint. A journal was collated by the first author, to record reflections on key points of interviews and to document the progression of code and theme development. Two volunteer participants reviewed and provided feedback on a written summary of results.

Quality can be affected by credibility, transferability, dependability, and confirmability. Each of these quality factors was considered as follows (Hannes, 2011). Credibility reflects whether the data represent the views of the participants and was assured by repeated analysis across time and person and by participants checking findings. Transferability evaluates whether findings are transferable to other settings. This was explored by understanding the demographics and context of participants. Dependability evaluates whether the research is logical, traceable, and clearly documented. In this study, it was supported through an audit trail of code and theme development supported by participants’ quotes. Finally, confirmability (or the extent to which the findings are grounded in the data) was supported by the description of the theoretical basis and methodological process, along with identification of the researcher’s backgrounds.

## Results

Initial thematic analysis led to 56 codes which were grouped into four themes. Two further iterations led to the identification of two overarching themes with five subthemes. Findings are described in Table 3. Italicized words are direct quotations from the mothers.

**Table 3.** Daily Life When Your Child Has SP-SI Differences: Themes, Subthemes, and Description With Example Quotes.

Themes and subthemes	Description	Example quotes
1. The impact on everyday life		
a. Changes in daily occupations	The child’s SP-SI differences have an impact on mother’s ability to participate in daily occupations	*It can be like walking on eggshells* (Eve) *She has really extreme reactions to self-care. Teeth . . . wash . . . hair . . . .nails it’s a fight even having breakfast* (Ceri) *Family days out I just wouldn’t do with all three of them. It wouldn’t be worth the risk* (Anne)
b. The things we have to do	Choices or actions that mothers have made as a direct consequence of their child or children’s SP-SI differences	*Both of the grandmas got concerned and they would try to put a spoonful of food into her mouth, but she never swallowed this food. She would retch, and I decided that I had to put a stop to this because it obviously wasn’t helping anything—I just hated it . . .* (Deb) *When we were choosing a house to move to, the criteria really was about how much noise she would be exposed to and things like that . . . Anything to get a good night’s sleep* (Anne)
c. A lack of knowledge and understanding	A lack of knowledge or understanding by others about everyday life for a mother of a child with SP-SI differences	*It’s the lack of understanding from other parents which is hard* (Bonnie) *I was made to believe that it was all behavioural because I was doing the wrong things as a parent . . . again, and again* (Ceri) *I would have liked a much greater awareness in the professionals including teachers and doctors* (Deb)
2. What helps?		
a. Empowering mothers: An attitude of understanding	Mothers are empowered when they and those who can support them and their child gain knowledge and understanding	*Once you understand a bit of why children are behaving the way that they are, it is much easier to manage.* *Thankfully school is very supportive* *My Mom, she kept backing me . . . she is a kind of lifeline* (Ceri) *I think the only thing about SPD is that it should be education, education, education for everybody . . . Because you need it* (Bonnie) *I found my voice, oh my . . . I didn’t stop* (Fiona)
b. Adaptation	Strategies and adaptations to daily life that support their participation in everyday life	*Finding the right toothpaste, that’s a game changer* (Fiona). *Soundproofing . . . wooden shutters . . . white noise maker improve it (sleep). . . . In the school holidays we go out every single day* (Anne) *We aim to have one of us here when our daughter comes in . . . It sounds crazy, she forgets to drink . . . I make sure the first thing I do is give her a drink* (Eve)

*Note*. SP-SI = sensory processing and sensory integration.

### Theme 1: The Impact on Everyday Life

This theme describes the experience of daily life for mothers of children with sensory processing differences.

#### Challenges in Daily Occupation

In the home, sleep, self-care, mealtimes, play, and chores were all identified as areas of challenge, with impacts on both child and mother. Deb shared her experience of both “meltdowns” at mealtimes—both from her and her child—“when (my) anger would come from nowhere.” Anne further reflected: “If there’s the slightest noise, she’s awake and then doesn’t go back to sleep. It . . . affects the whole family’s mood.” Unaddressed sensory needs place additional practical and emotional demands on mothers.

Mothers described the impact of sensory processing differences on their child’s role as a student including self-care. Fiona gave the example of her child’s difficulty in dealing with the sensory environment of the school toilets, which resulted in him avoiding the facilities all day, then rushing out and urinating outside school. She said: “They didn’t see it as a school issue. . .totally as a parenting problem.” Bullying was also identified as a problem in the school environment, resulting in a negative impact on social engagement.

Mothers also talked about restrictions to their own social, leisure and work opportunities. Difficulty coping with their child’s behavior led to avoidance of participation in community activities. Child safety concerns were highlighted, with a number of children running away from unanticipated or overwhelming sensory stimuli, for example, an ice-cream van (Bonnie) or a busy toy shop: “He put himself in so much danger” (Fiona). Safety was a factor in mothers’ decisions to participate or otherwise in shared leisure activities.

#### The Things We Have to Do

Mothers reflected on the challenges they faced in finding the right environment and/or support for their child. Support struggles ranged from minor (e.g., persuading school to allow a pencil topper) to extreme. One mother said,

We’d have meltdowns nearly everyday and I feel like we can’t live like this. We need to make some changes and I didn’t know how. So, I gave up my job. I sold my house and we moved to the coast. (Fiona)

Thus, not only are mothers making significant changes to their child’s environment and daily occupations but also to their own.

Additional resources needs were associated with raising a child with SP-SI differences. Parents “spend a fortune” (Eve) as well as time and emotional energy on “letters, reports and phone calls” (Deb). Where a child’s SP-SI differences have led to additional care or supervision requirements, mothers reported applying for government financial assistance, for example, Disability Living Allowance (DLA) (Bonnie). Unfortunately, despite meeting the criteria, it can be a challenge to access financial support:

It took a lot of effort and tears and formal complaint . . . It would have been much better if that kind of support had been more easily available. (Anne)

Mothers report that the impact of increased costs associated with meeting the needs of a child with SP-SI differences are exacerbated by difficulties in sustaining employment outside the home.

#### A Lack of Knowledge and Understanding

Mothers described their initial struggles to understand why their child was behaving in an atypical manner, before SP-SI differences were identified. Not understanding the reasons for their child’s “unusual” behavior left mothers frustrated:

She’d launch herself onto the sofa with a massive bounce and then her legs would be flying in front of you . . . it’s hard to refrain from shouting at the child for being in the way, when you just want a bit of downtime yourself. (Deb)

A lack of knowledge and understanding within family, social and professional networks impact how both mother and child are perceived. Reflecting back on her efforts to explain her child’s needs at school, Bonnie said: “It’s the lack of understanding that’s hard.” Deb described both grandmothers’ concerns over her child avoiding food, which led to criticism and a breakdown in these relationships. At times, the pattern of broken relationships extends beyond the family. Bonnie shared the experience of a community barbeque organizer who said: “I think it’s just naughty boy syndrome.” Bonnie’s response was to go elsewhere. Explaining, she said: “I just don’t want my son to be around (them) ‘cos they need education themselves.”

Inconsistent levels of knowledge, understanding and empathy from trained professionals were also reported by mothers. Moreover, pathways to identifying SP-SI differences were complex. Frequently, mothers were told their child’s behaviors were due to “bad parenting” (Ceri), with both health care and education staff showing a lack of awareness. Deb said: “it’s horrible not being believed” and said she reached the point where she felt “so angry at not being listened to . . . that there would be even more delay in getting my child what she needed.” The emotional impact on mothers was clearly expressed by Fiona when she said: “I didn’t know how to deal with it. I took it personally. I had lots of experts around me that laughed at me whenI suggetsed that there was something not quite right.” Both Deb and Eve expressed fears that reports or observations filed by third parties about their child’s sensory-related behavior would elicit child-protection concerns. Many mothers indicated that, as a consequence of poor understanding of their child’s behaviors, they felt isolated and alone.

### Theme 2: What Helps?

This theme explores factors that mothers said they found helpful in everyday life.

#### Empowering Mothers: An Attitude of Understanding and Support

An understanding and supportive attitude from family, school and the community empowers mothers. Describing the value of being listened to and understood, Deb said: “It was helpful to have an intelligent adult . . . our OT . . . listen to us about our child, take in what we were saying . . .not blanking us.”

Ann explained that a sensory profile completed by a nurse practitioner was “The most useful report we had ever read on her” and that it “ explained . . .behaviours . . . it was transformative for the whole family.” Being heard and understood, and gaining understanding, empower action and choice for mothers.

Mothers advocated for their own “social networks” (Anne) and “teachers” (Deb and Eve) having wider knowledge and understanding of the impact of SP-SI differences. Mothers identified both fathers (“Dads often get overlooked . .he’s very good” [Ann]) and grandmothers as key players in providing practical and emotional support to them. “If it wasn’t for my mum, I probably would have thought, uh, hang on,uh, I’m a really bad parent. . . she’s really supportive.” (Ceri)

An attitude of understanding, when it exists, supports valued participation by children with SP-SI differences. Bonnie describes how such an attitude helped her child in his leisure activity: “Thankfully we have an amazing . . .instructor – she gets him completely . . .she will be understanding in terms of what he needs to get in the zone.”

When the mother or child’s voice is heard, positive learning is gained. Ceri struggled with her daughter’s sensory-related issues after school meltdowns. School staff dismissed her concerns until a member of staff saw one such meltdown on the way home from school. After this, Ceri’s concerns were validated and her daughter was given a voice.

They actually said, when she left school, that they’d learned a lot from her…to develop their understanding of sensory: (Ceri)

Several mothers took direct action when they did not feel heard. Describing the steps she took, Fiona said: “Complain! . . .nothing changes unless you make those in the right places aware of what’s going wrong.”

Others actively searched for support and validation for themselves and their children. Bonnie said she felt very alone until she met “up with other parents”. Additional resources were cited as helpful, including books, social media groups, online resources, and support groups for parents, children, or teenagers.

When mothers and children are listened to, those around them gain knowledge and understanding. Helping family, friends, community leaders, education, and health care professionals be aware of their own value in supporting the child and the mother can lead to increased participation in everyday life for both.

#### Adapt

Adaptation of everyday occupations was described as an important way to support participation in daily life. In an unexpected example, mothers made adjustments to their infants’ breastfeeding habits to soothe a dysregulated child. “he was constantly (breast)feeding,” (Fiona). Often, this increased participation in breastfeeding also served the mother well, providing her with a period of calm. “Breastfeeding . . .gave me a kind of sensory reward . . I cannot imagine how I would have survivied parenting without (it).” (Deb).

Mothers also recognized the importance of adapting their environments, for example by providing a safe but challenging play space where, “you can move or crash about” or by establishing a “sound and light proofing bedroom” (Anne). Some families were able to adapt and tailor their routines to support the child, for example, by seeking out alternatives to homework (Eve). Physical activity was often seen as a key strategy, Anne reported that: “We are . . .an active family . . . we have to be to keep her regulated.”

Mothers can sensitively identify the impact of SP-SI differences, but it can remain challenging to adapt the activity or environment sufficiently. Potentially problematic situations are made easier when the parent understands their child’s preferences and responds accordingly, for instance, by knowing “what clothes to buy” (Anne). However, the best laid plans can be thwarted by factors that remain difficult, or even impossible, to control, such as the weather. “because of the heat, he could not tolerate clothes, we were housebound” (Fiona). Even in these situations, however, small adaptations can renew the possibility of participation, as one mother discovered when her child’s school showed some flexibility toward his sartorial needs. “He’s not tolerating school trousers – they’re quite lenient and he’s allowed to wear towelling shorts” (Fiona).

Access to intense outdoor physical activity was cited as important through either mainstream activities, such as gymnastics or through a specialist provision. Whether active or more relaxation-focused, leisure activities are appreciated by mothers for the value they bring their children with SI-SP differences. However, even when participation is possible and successful, accessing these opportunities brings an additional implications to mothers in terms of time, energy, and financial resources.

Over time, some mothers have been able to identify their child’s cues and to adapt an activity through preparation. An example of an area of adaptation was highlighted by Anne, Bonnie, and Fiona who all raised strategies to ensure “safety” in the community. Strategies included control of the sensory qualities of the environment, additional adult support and being able to attend activities at a quieter time (Ann, Bonnie, Fiona).

A successful application for government financial support (e.g., DLA) supports participation. Mothers not only use this funding for safety-related choices but also make decisions about how to spend the money based on what is meaningful to the family. Typical examples of how funds are spent include: additional childcare so the mother can work, fleece bedsheets, adapting toys, additional driving/swimming lessons, and multidisciplinary team meetings. Bonnie said: “I know we get DLA for both of them, and people feel guilty about claiming it, but I don’t for one minute because what I do spend the money on (is) these sorts of things.”

Activity/environmental adaptation and additional resources, both financial and emotional, provide valuable tools for mothers to support participation in everyday occupations for both of them and their children.

## Discussion

In mothers’ own voices, this study highlights the pervasive impact on everyday life of a child’s SP-SI differences on both the child and their mother. Mothers described their child’s SP-SI differences as presenting significant challenges for participation across all areas of occupation, including activities of daily living, work roles, and leisure activities at home, at school, and in the community. However, they also described the creative solutions they had found, including seeking out strong social networks and adapting everyday routines, even when these were at the expense of the mothers’ own work and leisure time.

These findings reflect an ecocultural perspective (Bronfenbrenner & Morris, 2006; Llewellyn, 2012) and how the mother and child interact with an environment that can act as a facilitator or a barrier to participation. At a microsystem level, mothers are helped by a supportive family network and the capacity to adapt both their environment and occupation. At an exosytem level, friends, school staff, health care staff, and community leaders with knowledge, understanding, and empathy support participation for both mother and child. At a wider mesosystems level, health, education, and social care policy influence accessibility to care, support, and financial opportunities. Occupational therapists have a role to play in direct intervention with the child and also in providing education to the communities and organizations around the child and family unit.

### Everyday Life

A child’s SP-SI differences create a barrier to participation in everyday life and increase caregiver strain with a changing pattern over time (Kirby et al., 2019). Our study further identifies the extensive practical and emotional experiences of everyday family life and illustrates the means by which mothers adapt their own occupations from the child’s infancy onwards. For example, at home, parents accommodate and adjust activities of daily living, such as dressing, sleep, and mealtimes. Outside the home, mothers negotiate the child’s needs at school. They also appear to face additional demands in the identification of, and access to, family leisure activities and in maintaining safety. Disruptions in daily occupations impact an activity and the secondary opportunities linked to it. For instance, shared mealtimes provide time for socialization and communication (Ochs & Shohet, 2006), however, where this occupation causes distress, the opportunity for positive interaction and social development is lost. Identification of such disruptions in daily life gives an opportunity for early intervention.

### What Helps?

#### Empowering Mothers

The experience of mothers in our study highlights how a lack of knowledge and understanding of SP-SI differences by both education and primary health care professionals impacts families. This has two implications. The first reflects an ongoing need to share information and offer education to our health care and education colleagues to facilitate development of knowledge, understanding, and empathy toward families. The second implication is that there are missed opportunities in reducing parental stress and enabling child/family participation in daily occupations. Mothers strongly identified the importance of being listened to and validated. Professionals who do not listen can hinder access to services and limit parents of disabled children in their decision-making capacity (Lundeby & Tossebro, 2008). In our study, mothers articulated the positive impact of an open and respectful dialogue with medical and educational professionals. The importance of listening has been raised at a policy level for autistic individuals in the United Kingdom, with specific focus on sensory sensitivities or overload (Pelicano et al., 2013). Mothers also strongly identified the importance of positive relationships across family, professional, and social networks in facilitating participation in daily activities. Showing respect and listening to individuals and families is at the heart of family-centered practice (Pozniak et al., 2023) and the importance of applying these principles to mothers of children with SP-SI differences is reinforced by the findings of our study.

Mothers in our study spoke of being judged and blamed, and this is reflected in other studies of mothers of children with SP-SI differences (Chiu, 2013) and more widely by parents of children with disability (Pozniak et al., 2023). The expectations placed on mothers act as a social barriers to participation. Conversely, social relationships can act as facilitators to participation. Our study uniquely reported the importance of fathers and grandmothers in providing both emotional and physical support, highlighting the value of developing positive relationships within social networks as an intervention strategy. Worth exploring is the potential of intervention practices that engage the wider family. Alternatively building a friendship/peer networks, such as occupational performance coaching groups (Suja Angelin et al., 2021). Another potential avenue for exploration is text-based communication networks for fathers (Ismael et al., 2018) .

The benefits, for both parents and society, of empowering parents are recognized in a study of parents’ experiences of advocating for their autistic child (Boshoff et al., 2016). Adult-learning theory suggests that providing information alone does not elicit change. The value of education with coaching is supported in studies with both individual parents of children with autism (Foster et al., 2013) and teachers of children with self-regulation issues (Hui et al., 2016). Coaching is a promising route to supporting the empowerment of mothers of children with SP-SI differences when these differences are impacting both mother and child’s participation in daily occupations.

### Adaptation

To support and improve participation, mothers highlighted the need to combine forward planning and adaptation of the environment. Adaptation of the environment has previously been identified to support participation in events, such as a community-based program to enhance access to museums (Silverman & Tyszka, 2017). As is central to occupational therapy practice (American Occupational Therapy Association [AOTA], 2020), the need to adapt the environment is valued by mothers across home, school, and community.

A notable support identified by mothers in this study was breastfeeding as a facilitator of maternal well-being and child self- or co-regulation. In typically developing infants, mother–child touch can reduce a mother's anxiety and improve her child’s behavioral and emotional outcomes (Pickles et al., 2016). In infants or mothers themselves (Talcer et al., 2021) who struggle to tolerate touch, typical regulatory strategies, such as hugs may not support co-regulation. The data presented suggest that breastfeeding provided a strategy to some dyads, as a means of both connection and co-regulation.

Mothers continuously adapt their own occupations to support the child, including by applying limitations to their own work roles. In particular, the challenge of supporting a child’s education means mothers are forced to reduce their work hours or even resign from employment outside the home. The ramifications of restricted access to employment and earnings are lifelong. In research on the financial impact on mothers of having an autistic child, it was found that earnings were 56% lower than for mothers of typically developing children (Cidav & Mandall, 2012). In our study, mothers were very clear about the value of additional government funding in supporting parent and child occupations that would otherwise be inaccessible.

## Limitations

Recruitment via specialist online parent forums identified mothers who were actively seeking out support or further information on the topic, future clinic-based recruitment may lead to a wider sample. The study focused only on mothers. To represent a wider population of caregivers, it would be necessary to look at all those who carry primary carer or shared carer responsibilities. In this small-scale study, participants were self-selecting and displayed limited social and ethnic diversity. Research with a more diverse populations is indicated. Most of the participants reported that their children had a diagnosis of autistic spectrum disorder. While it is difficult to fully separate the impact of a neurodevelopmental diagnosis from the impact of SP-SI differences, all participants attributed increased burden of care specifically to their child’s SP-SI differences.

## Conclusion

Mothers report that their child’s SP-SI differences impact daily occupations and social relationships. Mothers face additional demands to their parenting role from birth onwards, and advocate for early intervention. They adapt daily activities and their environments to meet the needs of their child and family, with varying levels of success. Mothers report that it is helpful when they and their children are listened to, when there is a supportive social and professional network, and when the activity and environment can be adapted to accommodate the child’s sensory needs. This study supports engaging with social and professional networks around the mother and child to promote knowledge and understanding of SP-SI differences and the impact these have across home, school and community settings.

## Supplemental Material

sj-docx-1-otj-10.1177_15394492241238357 – Supplemental material for Mothers’ Perspectives: Daily Life When Your Child Has Sensory Differences Supplemental material, sj-docx-1-otj-10.1177_15394492241238357 for Mothers’ Perspectives: Daily Life When Your Child Has Sensory Differences by Susan Allen, Amanda Branson, Shelly J. Lane and Fiona J. Knott in OTJR: Occupational Therapy Journal of Research

## References

[bibr1-15394492241238357] American Occupational Therapy Association. (2020). Occupational therapy practice framework: Domain and process fourth edition. American Journal of Occupational Therapy, 74, Article 410010. 10.5014/ajot.2020.74S2001 34780625

[bibr2-15394492241238357] Ben-Sasson A. Soto T. W. Martinez-Pedraza F. Cater A. S. (2013). Early sensory over-responsivity in toddlers with autism spectrum disorders as a predictor of family impairment and parenting stress. Journal of Child Psychology and Psychiatry, 54(8), 846–853. 10.1111/jcpp.12035 23336424 PMC3636173

[bibr3-15394492241238357] Booth A. Hannes K. Harden A. Harris J. Tong A. (2014). COREQ (Consolidated Criteria for Reporting Qualitative Studies). In Moher D. Altman D. G. Schulz K. F. Simera I. Wager E. (Eds.), Guidelines for reporting health research: A user’s manual (pp. 214–226). John Wiley &Sons.

[bibr4-15394492241238357] Boshoff K. Gibbs D. Phillips R. L. Wiles L. Porter L. (2016). Parents’ voices: ‘Why and how we advocate’—A meta-synthesis of parents’ experiences of advocating for their child with autism spectrum disorder. Child: Care, Health and Development, 42(6), 784–797. 10.1111/cch.12383 27445227

[bibr5-15394492241238357] Bowling A. (2014). Research methods in health: Investigating health and health services. Open University Press.

[bibr6-15394492241238357] Braun V. Clarke V. (2022). Thematic analysis: A practical guide. SAGE.

[bibr7-15394492241238357] Bronfenbrenner U. Morris P. A. (2006). The biological model of human development. In Lerner R. M. (Ed.), Handbook of child psychology: Theoretical models of human development (Vol. 1, pp. 297–242). John Wiley & Sons.

[bibr8-15394492241238357] Chiu E.-C. (2013). Preliminary study: Taiwanese mothers’ experiences of children with sensory processing disorder. The Journal of Nursing Research, 21(3), 219–223. https://doi.org/0.1097/jnr.0b013e3182a0afd4 23958612 10.1097/jnr.0b013e3182a0afd4

[bibr9-15394492241238357] Cidav Z. Mandall D. S. (2012). Implications of childhood autism for parental employment and earnings. Pediatrics, 129, 617–623. https://doi.org/0.1542/peds.2011-2700 22430453 10.1542/peds.2011-2700 PMC3356150

[bibr10-15394492241238357] Curtin M. Adams J. Egan M. (2017). Evolution of occupational therapy within the healthcare context. In Curtin M. Egan M. Adams J. (Eds.), Occupational therapy for people experiencing illness, injury or impairment: Promoting occupation and participation (7th ed., pp. 2–15). Elsevier.

[bibr11-15394492241238357] Dunstan E. Griffiths S. (2008). Sensory strategies: Practical support to empower families. New Zealand Journal of Occupational Therapy, 551(1), 5–13.

[bibr12-15394492241238357] Foster L. Dunn W. Lawson L. M. (2013). Coaching mothers of children with autism: A qualitative study for occupational therapy practice. Physical and Occupational Therapy in Pediatrics, 33(2), 253–263. 10.3109/01942638.2012.747581 23253014

[bibr13-15394492241238357] Hannes K. (2011). Critical appraisal of qualitative research. In Noyes J. Booth A. Hannes K. Harden A. Harris J. Lewin S. Lockwood C. (Eds.), Supplementary guidance for inclusion of qualitative research in Cochrane systematic reviews of interventions. (pp. 1–14). Cochrane Collaboration Qualitative Methods Group.

[bibr14-15394492241238357] Hui C. Snider L. Couture M. (2016). Self-regulation workshop and Occupational Performance Coaching with teachers: A pilot study: Étude pilote sur un atelier d’autogestion et des séances d’encadrement du rendement occupationnel à l’intention des enseignants. Canadian Journl of Occupational Therapy, 83(2), 115–125. 10.1177/0008417415627665 27026722

[bibr15-15394492241238357] Howitt D. (2010). Introduction to qualitative methods in psychology. Pearson Education.

[bibr16-15394492241238357] INVOLVE. (2012). Briefing notes for researchers: Involving the public in NHS, public health and social care research. https://www.invo.org.uk/wp-content/uploads/2014/11/9938_INVOLVE_Briefing_Notes_WEB.pdf

[bibr17-15394492241238357] Ismael N. Lawson L. M. Hartwell J. (2018). Relationship between sensory processing and participation in daily occupations for children with autism spectrum disorder: A systematic review of studies that used Dunn’s sensory processing framework. American Journal of Occupational Therapy, 72, 1–9. 10.5014/ajot.2018.024075 29689172

[bibr18-15394492241238357] Kirby A. V. Williams K. L. Watson L. R. Sideris J. Bulluck J. Baranek G. T. (2019). Sensory features and family functioning in families of children with autism and developmental disabilities: Longitudinal associations. American Journal of Occupational Therapy, 73(2), Article 205040. 10.5014/ajot.2018.027391 PMC6436113 30915965

[bibr19-15394492241238357] Law M. (2002). Participation in the occupations of everyday life, 2002 Distinguished Scholars lecture. American Journal of Occupational Therapy, 56, 540–649. 10.5014/ajot.56.6.640 12458856

[bibr20-15394492241238357] Llewellyn G. (2012). Ecocultural theory: Everyday cultures of children. In Bundy A. C. Lane S. J. (Eds.), Kids can be kids: A childhood occupations approach. (pp. 180–193). F. A. Davis.

[bibr21-15394492241238357] Lundeby H. Tossebro J. (2008). Exploring the experiences of ‘‘not being listened to’’ from the perspective of parents with disabled children. Scandinavian Journal of Disability Research, 10(4), 250–274. 10.1080/15017410802469700

[bibr22-15394492241238357] Miller-Kuhaneck H. Watling R. (2018). Parental or teacher education and coaching to support function and participation of children and youth with sensory processing and sensory integration challenges: A systematic review. American Journal of Occupational Therapy, 72(1), 1–11. 10.5014/ajot.2018.029017 29280713

[bibr23-15394492241238357] Ochs E. Shohet M. (2006). The cultural structuring of mealtime socialization. New Directions for Child and Adolescent Development, 111, 35–49. 10.1002/cd.154 16646498

[bibr24-15394492241238357] Parham L. D. Cosbey J. (2019). Sensory integration in everyday life. In Bundy A. C. Lane S. J. (Eds.), Sensory integration: Theory and Intervention (3rd ed., pp. 21–39). F. A. Davis.

[bibr25-15394492241238357] Pelicano L. Dinsmore A. Charman T. (2013). A future made together: Shaping autism research in the UK. University of London.

[bibr26-15394492241238357] Pickles A. Sharp H. Hellier J. Hill J. (2016). Prenatal anxiety, maternal stroking in infancy, and symptoms of emotional and behavioral disorders at 3.5 years. European Child & Adolescent Psychiatry, 26, 325–334. 10.1007/s00787-016-0886-6 27464490 PMC5323471

[bibr27-15394492241238357] Pozniak K. King G. Chambers E. Martens R. Earl S. de Camargo O. K. McCauley D. Teplicky R. Rosenbaum R. (2023). What do parents want from healthcare services? Reports of parents’ experiences with pediatric service delivery for their children with disabilities. Disability and Rehabilitation, 7, 1–14. 10.1080/09638288.2023.2229733 37419932

[bibr28-15394492241238357] Reynolds S. Glennon T. J. Ausderau K. Bendixen R. M. (2017). Using a multifaceted approach to working with children who have differences in sensory processing and integration. American Journal of Occupational Therapy, 71(2), e1–e7. 10.5014/ajot.2017.019281 PMC5317393 28218599

[bibr29-15394492241238357] Royal College of Occupational Therapists. (2021). RCOT informed view: Sensory integration and sensory-based interventions. https://www.rcot.co.uk/about-occupational-therapy/rcot-informed-views

[bibr30-15394492241238357] Royal College of Occupational Therapists. (2022). Children’s access to occupational therapy survey report. https://www.rcot.co.uk/occupational-therapy-children-and-young-people

[bibr31-15394492241238357] Schaaf R. C. Toth-Cohen S. Johnson S. L. Outten G. Benevides T. W. (2011). The everyday routines of families of children with autism: Examining the impact of sensory processing difficulties on the family. Autism, 15(3), 373–389. 10.1177/1362361310386505 21430016

[bibr32-15394492241238357] Silverman F. Tyszka A. C. (2017). Centennial topics—Supporting participation for children with sensory processing needs and their families: Community-based action research. American Journal of Occupational Therapy, 71, Article 100010. 10.5014/ajot.2017.025544 28661377

[bibr33-15394492241238357] Suja Angelin C. Sugi S. Rajendran K . (2021). Occupational performance coaching for mothers of children with disabilities in India. British Journal of Occupational Therapy, 88(1), 38–47. 10.1177/0008417420972868 33353385

[bibr34-15394492241238357] Talcer M. C. Duffy O. Pedlow K. (2021). A qualitative exploration into the sensory experiences of autistic mothers. Journal of Autism and Developmental Disorders, 53(2), 834–849. 10.1007/s10803-021-05188-1 34251566 PMC9944021

[bibr35-15394492241238357] Watling R. Miller Kuhaneck H. Parham L. D. Schaaf R. C. (2018). Occupational therapy practice guidelines for children and youth with challenges in sensory integration and sensory processing. American Occupational Therapy Association Press.

